# Stylasterid corals build aragonite skeletons in undersaturated water despite low pH at the site of calcification

**DOI:** 10.1038/s41598-022-16787-y

**Published:** 2022-07-30

**Authors:** Joseph A. Stewart, Ivo Strawson, James Kershaw, Laura F. Robinson

**Affiliations:** 1 School of Earth Sci. Univ. of Bristol, Queens Road, Bristol, BS8 1RJ UK; 2 Department of Earth Sciences, University of Cambridge, Downing Street, Cambridge, CB2 3EQ UK

**Keywords:** Marine biology, Climate change, Palaeoceanography, Climate-change ecology, Marine chemistry

## Abstract

Anthropogenic carbon emissions are causing seawater pH to decline, yet the impact on marine calcifiers is uncertain. Scleractinian corals and coralline algae strongly elevate the pH of their calcifying fluid (CF) to promote calcification. Other organisms adopt less energetically demanding calcification approaches but restrict their habitat. Stylasterid corals occur widely (extending well below the carbonate saturation horizon) and precipitate both aragonite and high-Mg calcite, however, their mode of biocalcification and resilience to ocean acidification are unknown. Here we measure skeletal boron isotopes (δ^11^B), B/Ca, and U/Ca to provide the first assessment of pH and rate of seawater flushing of stylasterid CF. Remarkably, both aragonitic and high-Mg calcitic stylasterids have low δ^11^B values implying little modification of internal pH. Collectively, our results suggest stylasterids have low seawater exchange rates into the calcifying space or rely on organic molecule templating to facilitate calcification. Thus, despite occupying similar niches to Scleractinia, Stylasteridae exhibit highly contrasting biocalcification, calling into question their resilience to ocean acidification.

## Introduction

At current anthropogenic carbon emission rates, surface seawater pH is predicted to fall below 7.8, and carbonate saturation states (Ω = [Ca^2+^] × [CO_3_^2−^]/K^*^_sp_) will greatly reduce before the end of this century^1^. Such large-magnitude and (geologically) rapid ocean acidification is expected to be a key stressor for marine calcifying organisms^2^, with many studies showing a reduction in stony coral health, abundance, and calcification rates under low seawater pH conditions (e.g.^3^). Yet without detailed knowledge of coral biocalcification mechanisms and adaptive strategies the full impact of ocean acidification on these marine calcifiers remains unclear. Particular concerns have been raised as to the vulnerability of deep-sea corals that remain poorly studied and occupy niches that are likely to be at the limits of their carbonate saturation tolerance already^4,5^.

Mineralogy plays an important role in dictating the susceptibility of carbonate to dissolution^6,7^. Calcite is a relatively dissolution-resistant form of CaCO_3_, typically adopted by unicellular foraminifera^8^. Aragonite is a more soluble polymorph, yet many organisms, including scleractinian corals, use this mineral^9^. As more magnesium is incorporated into calcite (up to ~ 140 mmol/mol Mg/Ca) its solubility increases towards that of aragonite^7^. Many organisms calcify using this intermediate solubility “high-Mg” calcite including Isidiid gorgonian “bamboo” corals^10^ and sea urchins^11^. As the Mg content of calcite increases further (> 140 mmol/mol Mg/Ca) the solubility of this high-Mg calcite falls below even that of aragonite. Organisms that adopt this more soluble mineral form to build their skeletons include crustose coralline algae^12^.

With a diverse array of carbonate minerals used by marine organisms, different calcification pathways are required to construct and maintain these skeletons. However, there have been comparatively few studies where microsensors or pH sensitive dyes have been placed within living biocalcifiers to monitor pH at the site of calcification as they require sophisticated laboratory culture setup and cannot be applied to coral specimens post-mortem (e.g.^13–15^). Skeletal geochemistry is therefore the go-to approach for understanding the mineralisation process, with the boron isotopic composition (δ^11^B; ^11^B/^10^B ratio relative to the standard NIST SRM 951 in ‰) of marine carbonates shown to be a powerful tool to assess pH at the site of calcification^16,17^.

**Figure 1:**
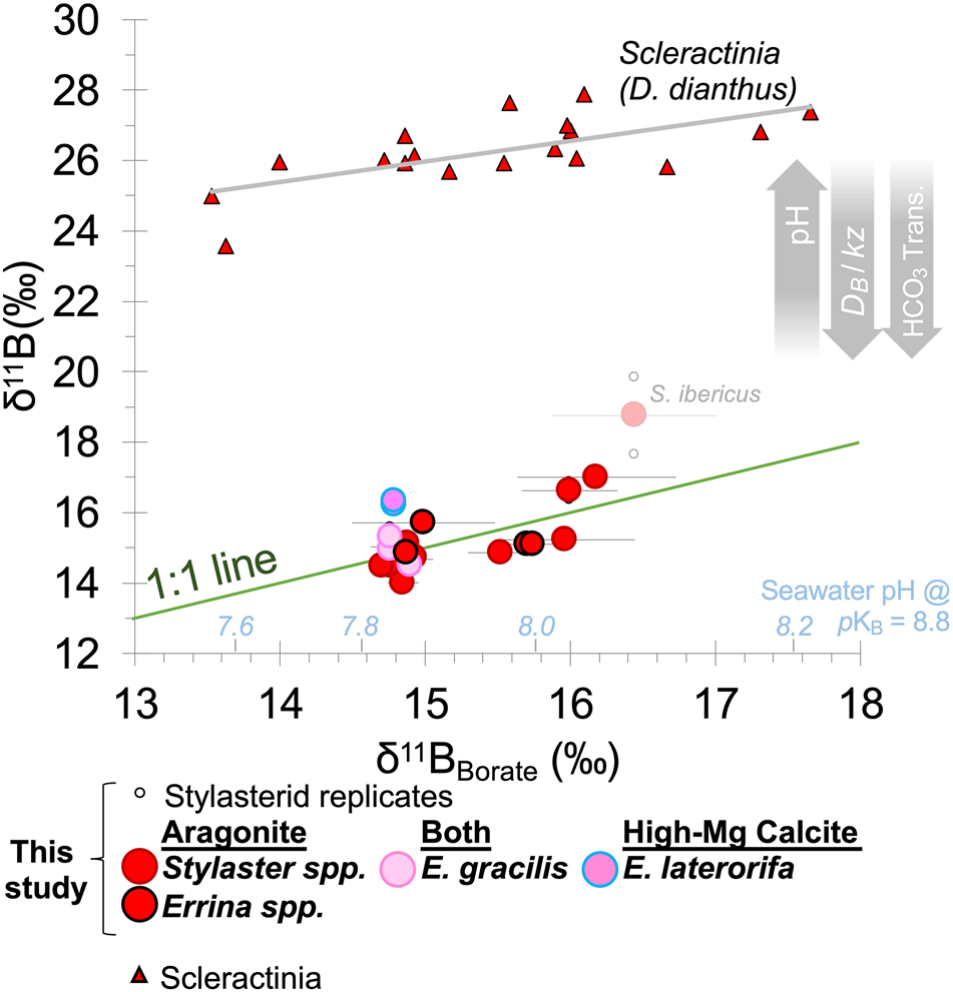
Coral δ^11^B plotted against δ^11^B of the borate ion in seawater (a function of pH; blue labels). Stylasterid corals (circles; this study) are compared to the well-characterised aragonitic scleractinian *Desmophyllum dianthus*^16,30,31^ that strongly upregulate pH at the site of calcification. *D. dianthus* replicates are averages and one shallow water fjord sample from McCulloch et al.^16^ was excluded because of high seawater pH variability at that site. Error bars denote the estimated uncertainty on seawater borate δ^11^B.

**Figure 2:**
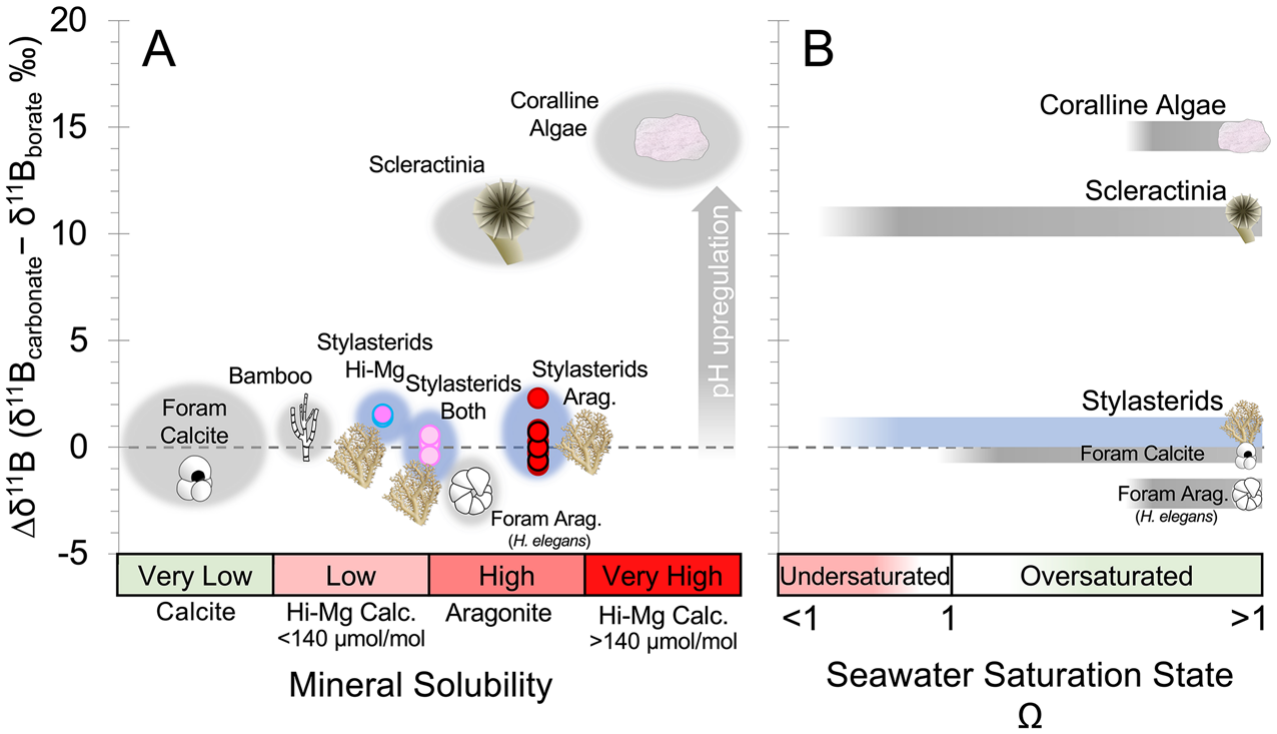
Skeletal carbonate δ^11^B offset from δ^11^B of borate in seawater (∆δ^11^B)—a measure of calcifying fluid pH upregulation—compared to (**A**) mineralogy and (**B**) carbonate ion saturation of seawater (Ω) habitat. Stylasterid coral ∆δ^11^B are compared to published δ^11^B values for aragonitic scleractinia^16,30,31^, high-Mg calcitic bamboo corals^32^, calcitic and aragonitic foraminifera^35^, and crustose coralline algae^18,21,22^. Mineral solubility from^7^.

Scleractinian corals and coralline algae adopt more dissolution-susceptible mineral forms, but calcify from fluids that are semi-restricted from ambient seawater and in which pH is biologically elevated to promote calcification. This “pH upregulation” varies by taxon, but in cold-water Scleractinia it is ~ 1 pH unit above ambient seawater^16^. This strong elevation of pH in the CF results in skeletal δ^11^B values > 8‰ higher than seawater borate values (Fig. 1; high ∆δ^11^B; where ∆δ^11^B = δ^11^B_carbonate_ – δ^11^B_borate_; Fig. 2)^16,18–22^. While photosynthetic coralline algae are restricted to the photic zone with its generally higher carbonate saturation states, the adaptation of pH upregulation has enabled azooxanthellate cold-water Scleractinia to live much deeper, even in undersaturated seawater conditions (Fig. 2^5^). It has been suggested that biogenic aragonites that lack strong pH upregulation strategies (resulting in low ∆δ^11^B values) such as the benthic foraminifera *Hoeglundina elegans* have a more restricted habitat and only calcify when ambient seawater is oversaturated with respect to aragonite, in areas such as the shallow Bahamas platform (Fig. 2^23^). Many studies have suggested that the increased buffering associated with pH upregulation will make Scleractinia resilient against moderate changes in ocean pH^16,24^, however others continue to highlight the potential vulnerability of these corals in the deep sea and question the energetic demands of this biocalcification strategy^4,5,25^.

Stylasteridae (Class Hydrozoa: Order Anthoathecata) are the second most diverse group of hard corals in the global ocean^26,27^. The majority of stylasterid species can be found below 50 m water depth making this an important, but poorly studied, deep-water coral family^26,27^. These Hydrozoan corals differ significantly from scleractinian corals in their ability to precipitate skeletons of either aragonite, high-Mg calcite, or in some cases, both mineral polymorphs^26^. Compared to Scleractinia, skeletal δ^18^O and δ^13^C in stylasterid corals are close to equilibrium with seawater^28^, however a positive covariation of these δ^18^O and δ^13^C ratios suggests that there may be at least some biological modification of the internal seawater carbonate chemistry^28^. Contrary to expectation, the stylasterid corals that deviate least from equilibrium δ^18^O and δ^13^C values were found to be aragonitic specimens (~ − 3‰) implying that there is less modification of internal carbonate chemistry than high-Mg calcitic specimens, despite their greater mineralogical vulnerability to dissolution^28^. While this may represent a mineralogical control on skeletal stable isotope values, little is known about how stylasterids biomineralize and are able to thrive in undersaturated seawater conditions.

**Figure 3:**
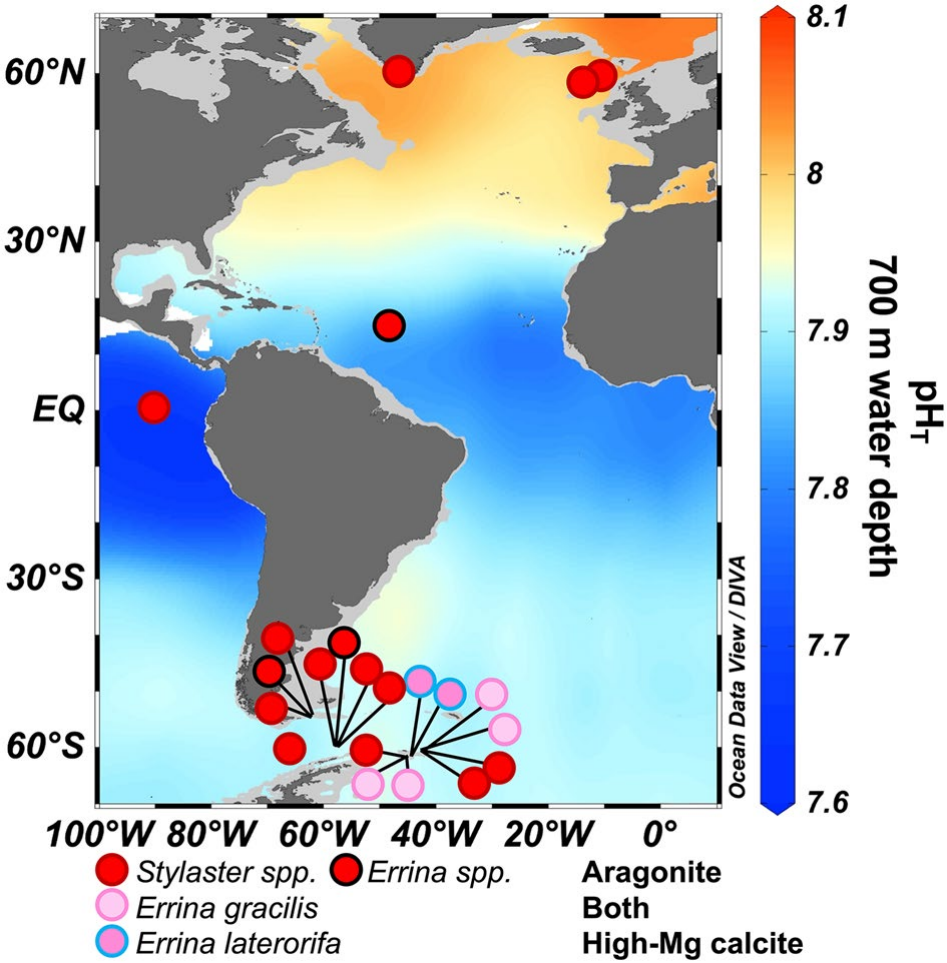
Location of Stylasteridae coral samples used in this study. Base map shows GLODAP v2 gridded seawater pH (total scale) at 700 m^65^. Map drawn using Ocean Data View software (Version 4.6.5; https://odv.awi.de/)^72^.

To address the gap in understanding of stylasterid coral biocalcification, we present the first skeletal δ^11^B, B/Ca, and U/Ca data obtained from stylasterid coral skeletons (Fig. 3). The samples in this study lived in seawater environments ranging from pH 7.86 to 8.04 (Ω_Arag._ 0.9 to 2.0). They comprise two genera of stylasterid corals (*Stylaster* and *Errina*) that represent all three modes of stylasterid skeletal mineralogy: (i) solely aragonite, (ii) both aragonite and high-Mg calcite, and (iii) solely high-Mg calcite^28,29^. We use the same stylasterid specimens for which skeletal stable oxygen and carbon isotopes have been previously characterised^28^ for comparison with our carbonate system proxy results.

## Results

### Stylasterid boron isotopes

Replicate measurements of bulk stylasterid δ^11^B of the same individual were generally within 0.5‰ of each other (Fig. 1). Once δ^11^B replicates for each stylasterid specimen are averaged, mean δ^11^B values are typically within 1‰ of the estimated δ^11^B composition of borate in ambient seawater for each coral (Fig. 1). The only samples (excluding *Stylaster ibericus*) with mean δ^11^B values more than 1‰ higher than δ^11^B_borate_ were the high-Mg calcitic *E. laterorifa* (δ^11^B = δ^11^B_borate_ + ~ 1.5‰). We therefore find little appreciable difference in δ^11^B between high-Mg calcitic and aragonitic stylasterid corals that live in similar seawater pH conditions. The single *Stylaster ibericus* specimen in this study yielded anomalously high δ^11^B (and low B/Ca) compared to other aragonitic stylasterids. This is discussed in detail in the Supplementary Information; however, its inclusion here has little impact on our overall findings.

We perform linear regression of stylasterid δ^11^B measurements against δ^11^B_borate_. The following relationships include all stylasterid δ^11^B measurements (regardless of mineralogy; Eq. 1), aragonitic stylasterids only (Eq. 2), and weighted regression of aragonitic stylasterids (Eq. 3), against δ^11^B_borate_ (SE):

1 $$\delta^{{{11}}} {\text{B}}_{{{\text{Stylasterids All}} }} = { 1}.{28 \,}\left( {0.{33}} \right) \, \times \, \delta^{{{11}}} {\text{B}}_{{{\text{borate}}}} {-}{ 4}.{1}0 \, \left( {{4}.{96}} \right) \left[ {{\text{R}}^{{2}} = \, 0.{43}} \right]$$

2 $$\delta^{{{11}}} {\text{B}}_{{{\text{Stylasterid Arag}}. }} = { 1}.{57\, }\left( {0.{33}} \right) \, \times \, \delta^{{{11}}} {\text{B}}_{{{\text{borate}}}} {-}{ 8}.{66\, }\left( {{5}.0{5}} \right) \left[ {{\text{R}}^{{2}} = \, 0.{62}} \right]$$

3 $$\delta^{{{11}}} {\text{B}}_{{{\text{Stylasterid Arag}}.{\text{ WT}} }} = { 1}.0{4 }\left( {0.{29}} \right) \, \times \, \delta^{{{11}}} {\text{B}}_{{{\text{borate}}}} {-} \, 0.{72 }\left( {{4}.{34}} \right) \left[ {{\text{R}}^{{2}} = \, 0.{49}} \right]$$

The regression in Eq. (3) weights observations according to the inverse of the uncertainty on their mean skeletal δ^11^B measurements. For this, the range in δ^11^B of the two replicate analyses about the mean was used. In instances where this range was less than analytical uncertainty, a value of ± 0.16‰ was used instead. These equations open the possibility to utilise stylasterid corals as archives for past seawater pH reconstruction (Supplementary Information).

### Stylasterid B/Ca and U/Ca

**Figure 4:**
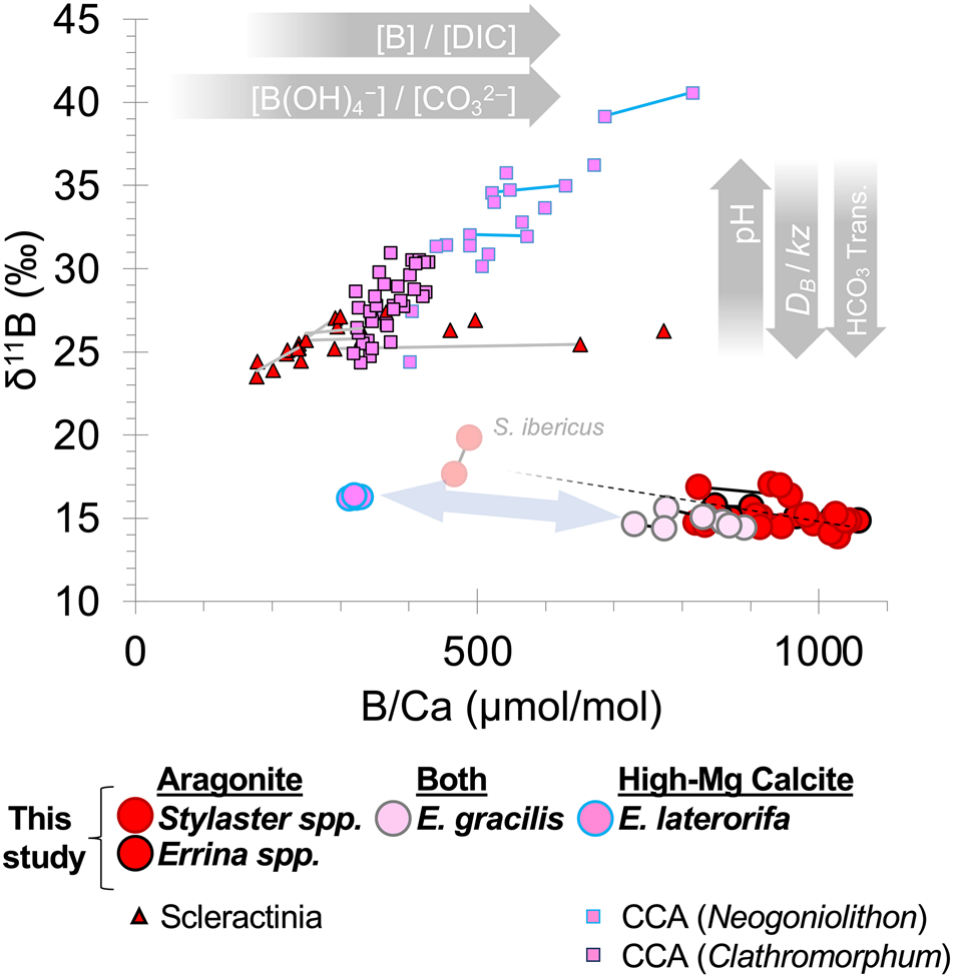
Carbonate B/Ca against δ^11^B. Stylasterid B/Ca and δ^11^B replicate results (this study) are compared to fibrous aragonite within scleractinia (*D. dianthus *)^47^, and crustose coralline algae (CCA^18,21^). Individual analyses from the same specimens are connected by solid trend lines (2 analyses or more). Dashed trendline shows the covariance between all aragonitic stylasterid measurements (p < 0.01). Blue arrow shows theoretical mixing line between solely aragonitic and solely high-Mg calcitic stylasterids.

**Figure 5:**
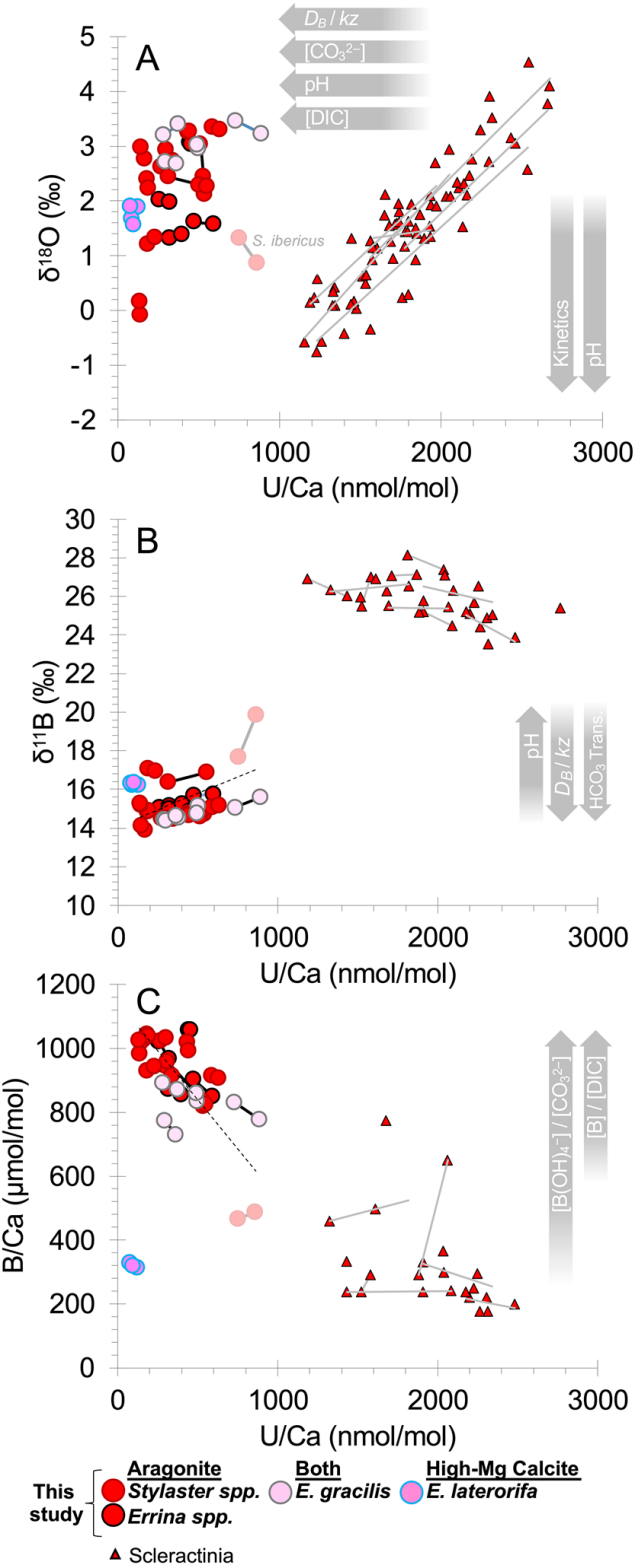
Coral U/Ca against (**A**) δ^18^O and (**B**) δ^11^B (**C**) B/Ca. Stylasterid U/Ca, B/Ca, and δ^11^B data are from this study, paired with δ^18^O measurements in the same specimens^28^. Scleractinian *D. dianthus* data are from micro-sampled specimens by Chen et al.^53^ and Stewart et al.^47^. Individual analyses from the same specimens are connected by solid trend lines (2 analyses or more). Dashed trendlines show the covariance between all aragonitic stylasterid measurements (p < 0.01).

B/Ca replicate measurements made on individual pieces of stylasterid coral were typically within 7% for each specimen. U/Ca measurements were considerably more variable between replicates, on average differing by 14% for both aragonitic and calcitic specimens. Mean B/Ca and U/Ca values for high-Mg calcitic specimens were generally lower (< 330 and < 110 µmol/mol respectively) than aragonitic stylasterids (average 918 and 392 µmol/mol respectively) (Figs. 4 and 5). B/Ca and U/Ca results for the mixed mineralogy species *E. gracilis* lie on a mixing line between pure aragonitic specimens and high-Mg calcitic specimens, though falling closest to the pure aragonites, consistent with their 92 to 96 weight percent aragonite content^28^ (Figs. 4 and 5).

Where there were differences between B/Ca and δ^11^B duplicate measurements (i.e. > 0.2‰; n = 6; excluding *S. ibericus*), in all cases B/Ca was found to be negatively correlated with δ^11^B for both aragonitic and mixed mineralogy specimens (Fig. 4). U/Ca values in aragonitic stylasterids show little covariance with previous δ^18^O measurements in the same specimens (Fig. 5A; ^28^), but positive covariance with skeletal δ^11^B measurements (Fig. 5B), and slight negative covariance with B/Ca (Fig. 5C). These generalised trends hold both between specimens and between sample replicates (Figs. 4 and 5).

## Discussion

The most important result in this study is the low skeletal δ^11^B measured in all stylasterid corals (Fig. 1) with no apparent offset from ambient seawater δ^11^B_borate_ (low ∆δ^11^B; Fig. 2). These results are in stark contrast to high ∆δ^11^B values recorded in scleractinian corals^16,30,31^ and coralline algae^18,21,22^ known to strongly modify pH at the site of calcification to promote mineral growth (Fig. 2). Instead, the low ∆δ^11^B values we record in stylasterid corals are more similar to bamboo corals^32^, sea urchins^11,33^, bivalves^33,34^, and unicellular foraminifera (e.g. *Cibicidoides wuellerstorfi* or *H. elegans*)^35^ (Fig. 2). However, many of these organisms are composed of dissolution-resistant mineral forms (e.g. calcite). Stylasterids on the other hand, while not the first aragonitic taxon to exhibit low skeletal δ^11^B (e.g. *H. elegans*), are the first to show this feature whilst living at depths close to and below the carbonate saturation horizon^36^. Assuming that stylasterid δ^11^B reflects the pH of the CF according to the abiotic relationship between the borate ion and pH (Eq. (4); Methods; e.g.^16,17^), these δ^11^B results suggest no modification of internal seawater pH in stylasterids. Furthermore, application of a physiochemical biocalcification model based on scleractinian corals by DeCarlo et al.^37^ (Methods), the high B/Ca values in our aragonitic stylasterids imply low [CO_3_^2−^] in the CF (average 110 µmol/kg excluding *S. ibericus*) similar to external seawater (average 90 µmol/kg). Low [CO_3_^2−^] in the CF would in turn translate to low CF Ω_Aragonite_ (average 1.5 excluding *S. ibericus*; 4 corals have Ω_Aragonite_ < 1). This contrasts with similar modelling of δ^11^B and B/Ca data in scleractinian corals which suggest CF Ω_Aragonite_ is typically more than 5 times that of ambient seawater^38^. Although our estimates for stylasterid CF Ω_Aragonite_ represent minimum values (i.e. no transport of Ca^2+^ to the CF), it remains hard to explain how stylasterids commence calcification at all at such low saturation states. Like stylasterid corals, the δ^11^B of many foraminifera record the δ^11^B of borate and thus external seawater pH^35,39^, however microsensor studies show the pH of foraminiferal CF is actually slightly elevated (+ 0.5 pH units)^40^. We therefore explore alternative mechanisms that may facilitate mineralisation at low Ω and/or decouple the boron skeletal chemistry from calcification processes including: (i) bicarbonate active transport^41^, (ii) high boric acid to seawater flushing rates^25^, and (iii) organic molecule templating^42^.

Despite cold-water scleractinian corals strongly upregulating pH, unusually low δ^11^B values (8.5‰) have been observed in their centres of calcification (or early mineralisation zones), particularly in *Lophelia pertusa*^41,43,44^. This disconnect between the internal pH and skeletal δ^11^B has been explained by bicarbonate (HCO_3_^−^) active transport to the site of calcification^41^. The use of HCO_3_^−^ as a source of DIC rather than CO_2_ (from respiration and diffusion) promotes mineralisation as it releases fewer protons during conversion to CO_3_^2−^. The bicarbonate active transport model suggests that seawater borate is co-transported to the site of calcification, resulting in low skeletal δ^11^B values closer to seawater borate^41^. While this mechanism could explain low δ^11^B values in stylasterid corals, studies of the scleractinian coral *Lophelia pertusa* suggest that these centres of calcification are also associated with low B/Ca compared to the bulk coral (− 40%)^41,44^. This contrasts with the extremely high B/Ca values we observe in our aragonitic stylasterid corals compared to Scleractinia, suggesting an alternative mechanism is driving their boron skeletal chemistry.

Another approach to reconciling discrepancies between pH derived from microsensors and geochemistry comes from coral modelling work by Gagnon et al.^25^. In this model, internal scleractinian pH is held at a high, but constant, value of 8.9 (resulting in their characteristic high ∆δ^11^B) and low skeletal δ^11^B values are instead explained by higher rates of boric acid diffusion (*D*_*B*_) relative to seawater exchange in the CF (*kz*; where *k* is the rate of seawater exchange and *z* is the volume to surface area ratio of the CF); where *D*_*B*_/*kz* scales linearly with seawater dissolved inorganic carbon ([DIC]). High *D*_*B*_/*kz* may therefore explain the low δ^11^B values in stylasterid corals, with low seawater flushing rates allowing them to occupy hostile growth environments (e.g. Ω_Aragonite_ as low as 0.5 (depths > 500 m) in the high latitude North Pacific^45^). To explore this hypothesis further, we use U/Ca and B/Ca ratios, with their sensitivity to the carbonate chemistry of seawater to test the potential role of *D*_*B*_/*kz* in stylasterids.

A positive correlation between carbonate δ^11^B and B/Ca is readily explained because at high pH more of the boron is in the borate ionic form and readily incorporated into the carbonate lattice^46^. This phenomenon has been observed in scleractinian corals^47^ and crustose coralline algae^18,21^ (Fig. 4). However, we find the opposite—a negative correlation between δ^11^B and B/Ca in sample replicates of stylasterid corals and particularly high B/Ca ratios in aragonitic specimens which cannot be explained by this effect. Although boron is preferentially incorporated into an aragonite lattice over calcite ^48^, this cannot account for the near double skeletal boron concentration in aragonitic stylasterids compared to scleractinian corals with the same mineralogy. While this may imply low concentrations of [DIC] and/or [CO_3_^2−^] (e.g. DeCarlo et al.^37^ model (Methods)), an alternative explanation for high B/Ca ratios and the negative correlation between δ^11^B and B/Ca in stylasterid corals is high *D*_*B*_/*kz*. Higher rates of boric acid diffusion relative to seawater exchange would simultaneously drive δ^11^B low whilst increasing [B] in the CF, thus driving B/Ca high (i.e. high [B]/[DIC]^49^).

The pH and/or [CO_3_^2−^] dependency of uranyl ion complexation in seawater gives a strong theoretical basis for the sensitivity of coral U/Ca to the carbonate system^50,51^. The master variable however is not clear and many potential drivers of coral U/Ca have been suggested including: inverse relationships with (i) calcifying fluid [CO_3_^2−^]^50,52,53^, (ii) pH^53,54^, and (iii) [DIC]^55^, as well as (iv) positive correlation with the rate of seawater replenishment of the coral calcifying space (*kz*)^55^. Evidence for the pH effect on U/Ca comes from positive covariance between δ^18^O and U/Ca^53^ (Fig. 5A) and negative covariance between δ^11^B and U/Ca^47^ (Fig. 5B) measured in replicate samples of scleractinian corals that strongly upregulate pH at the site of calcification. Thus while covariation of δ^18^O and δ^13^C ratios suggests perhaps some modification of internal pH^28^, the absence of correlation between δ^18^O and U/Ca (either between stylasterid coral specimens or between replicates) suggests that any pH upregulation in stylasterids is small enough not to impact U/Ca (Fig. 5A). Stylasterid coral results further contrast with scleractinian corals by exhibiting a positive correlation between δ^11^B and U/Ca between sample replicates (Fig. 5B). This is a counterintuitive result in the context of the pH and [CO_3_^2−^] effects on coral U/Ca ratios (e.g.^53^), however if *D*_*B*_/*kz* were the main driver of stylasterid coral U/Ca (e.g.^55^) then the positive covariance between δ^11^B and U/Ca can be reconciled (Fig. 5B) along with low U/Ca values that negatively correlate with B/Ca (Fig. 5C). Thus, high *D*_*B*_/*kz* can explain both the boron and uranium skeletal chemistry of stylasterids, with any small degree of biologically induced pH increase at the site of calcification (required to facilitate boric acid diffusion) potentially masked by low seawater flushing rates.

The mechanisms discussed so far have largely focussed on physiochemical processes inside a coral where mineralisation (ion-by-ion attachment) is dictated by Ω of the CF and where the elemental skeletal chemistry is driven by growth rate effects. Recent studies however highlight the important role that coral organic molecules may play in biocalcification (e.g. review by Drake et al.^42^). Acid-rich proteins in corals can spontaneously catalyse the formation of amorphous CaCO_3_^56^. Under this biologically mediated framework, the calcifying space requiring manipulation is just nanometers in size, comprising vesicles that both bind Ca^2+^ and transport the amorphous CaCO_3_ to the site of deposition^42^. This mechanism yields elemental skeletal chemistry which is more dependent on coral physiology^42^. The contention that stylasterid mineralogy (aragonite or high-Mg calcite) is more dictated by genetics rather than environmental parameters (e.g. seawater Ω_Aragonite_)^29^ does speak for a strong biological control on calcification. Thus, while aragonite may be vulnerable to dissolution^7^, it is potentially the more thermodynamically favourable mineral to form when mediated by acid-rich proteins in corals from modern seawater^56^. A reliance on organic matrix templates for mineralisation may therefore explain the paradoxically low skeletal δ^11^B values in stylasterids given their depth habitat. The presence of seawater at the site of calcification would also result in low skeletal δ^11^B values directly linked to ambient seawater pH. A strong biological control on calcification would therefore not preclude the use of stylasterid δ^11^B for use as a tracer for past seawater pH (e.g. Eq. (1); Supplementary information).

The energetic burden of calcifying via biologically-controlled organic molecule templating is considered low, such that it may confer resilience to organisms against future changes in ocean pH^56^. However, many of the low pH and undersaturated habitats in which stylasterid corals appear to thrive are on seamounts^45^. These submarine features, particularly those at high-latitude, are often areas of localised current variability (suitable for larval dispersion), high primary production, and high organic flux to the seafloor (food supply), resulting in biodiversity hot-spots^57^. A ready source of metabolic energy is important in many marine calcifiers (e.g.^14^), therefore it is possible that stylasterid corals compensate for low saturation conditions with additional metabolic energy sourced from an abundant food supply in these otherwise seemingly inhospitable environments. Some resilience to low pH waters may also come from their relatively thick layer of organic tissue that has been shown to shield the skeletons of other corals from undersaturated seawater^58^.

## Conclusions

Stylasterid corals are of great ecological importance, occupying niches that other corals do not, thus providing vital habitat for other benthic organisms^26,59^. Understanding and mitigating the impacts of climate change on these corals is therefore key to their conservation. The low skeletal δ^11^B values that we record in all stylasterid corals, regardless of their mineralogy, closely resemble seawater δ^11^B_borate_ suggesting that any biological upregulation of pH at the site of calcification in stylasterids is small. This style of biocalcification contrasts greatly with other aragonitic deep-water biocalcifiers (e.g. Scleractinia) and may be a result of an entirely different evolutionary history. For instance, many benthic marine fauna are thought to have originated in shallow waters and evolved to fill deep-water niches, whereas stylasterids are thought to have undergone an opposing, offshore to onshore, habitat expansion^60^. We argue that the low δ^11^B and generally high B/Ca and low U/Ca values we record in aragonitic stylasterids are best explained by (i) lower seawater exchange rates and/or higher rates of boric acid diffusion into the CF compared to scleractinian corals or (ii) a reliance on organic matrix templating for calcification.

Without a strong internal pH upregulating mechanism, physiochemical models of biocalcification would predict that stylasterids precipitating aragonite will be more vulnerable to future declines in seawater pH than those that have adopted the more dissolution-resistant high-Mg calcitic mineral form. However, if their calcification is strongly biologically mediated, this strategy may prove advantageous over other taxa which rely on ion pumping to modify their calcifying fluid chemistry (e.g. scleractinian corals). Determining the energetic burden of their calcification strategy will therefore be key to understanding the resilience of stylasterids to future change.

## Methods

### Stylasterid samples

Stylasterid samples were collected for this study from the Labrador Sea, (RRS *Discovery* DY081), Northeast Atlantic (RRS *James Cook* JC136), Equatorial Atlantic (RRS *James Cook* JC094), Drake Passage (R/V *Nathaniel B. Palmer* 1103 and 0805; RRS *James Clark Ross* JR15005), Galapagos Archipelago (*Alucia* Cruise AL1508) and (Supplementary Information). With the exception of two individuals (which appear to have “pristine” preservation based on visual inspection), all samples in this study were collected alive, with associated organic tissue, thus ensuring paired hydrographic data are representative. Stylasterid samples are grouped by mineralogy (i) solely aragonite (*E. antarctica, E. boschmai, E. altispina, S. robustus, S. densicaulis, S. erubescens, S. ibericus, S. marenzelli*; Mg/Ca ~ 2.5 mmol/mol), (ii) both aragonite and high-Mg calcite (*E. gracilis*; Mg/Ca ~ 12 mmol/mol), and (iii) solely high-Mg calcite (*E. laterorifa*; Mg/Ca ~ 90 mmol/mol)^28,29,61^. The Li/Mg and Sr/Ca ratios of these same specimens were previously measured by Stewart et al.^61^. Because *E. laterorifa* Mg/Ca values are less than 140 µmol/mol, the high-Mg calcitic corals in this study are considered theoretically more resistant to dissolution than pure aragonitic counterparts based solely on their mineralogy^7^.

### Matching hydrographic data

To characterise ambient seawater conditions at each site we use the same paired hydrographic data to that of Stewart et al.^61^. Alkalinity and [DIC] measurements were used in conjunction with temperature, salinity, and nutrient data to calculate full carbonate system parameters at each location including pH (total scale); Seacarb package in R^62^). This calculation used carbonate dissociation constants from Lueker et al.^63^ and the boron to salinity ratios of Lee et al.^64^. Where possible seawater hydrographic data were taken from co-located remotely operated vehicle or CTD (conductivity, temperature, and depth) profiles taken during the respective research cruise (10 samples from cruises DY081, JC094, NBP1103, and NBP0805). Where paired shipboard data were unavailable results from nearby bottle data in the GLODAP v2 database were used^65^. Hydrographic stations were typically within ~ 150 km of coral sampling sites and 30 m depth of the samples. The maximum offset between corals and water data was at the Galapagos site (530 km distance and 89 m water depth) owing to the poor regional sampling coverage. To assess uncertainty on pH estimates caused by both true in-situ variability and distance between coral and hydrographic stations, a further two nearby bottle seawater measurements were selected from the combined GLODAP v2 and research cruise databases (Supplementary Information). For most sites this variability was within ± 0.04 pH units (2σ) with a maximum variation of ± 0.09 pH units. At sites where pH was more variable the most proximal bottle data is still preferred over averaging all three pH estimates.

### δ^11^B of borate in seawater (δ^11^B_borate_)

Both the concentration and δ^11^B of the borate ion (δ^11^B_borate_) increase as a function of seawater pH^46,66,67^. The incorporation of this charged ion into marine calcium carbonates as they precipitate therefore forms the premise of the boron isotope pH-proxy^66^. Here we calculate δ^11^B_borate_ of ambient seawater experienced by each coral using the seawater pH estimates above and the simplified equation (Eq. 4) rearranged from Zeebe and Wolf-Gladrow^67^:

4 $${\updelta}^{11}{{\text{B}}}_{\text{borate}}=\frac{{\updelta}^{11}{{\text{B}}}_{\text{sw}} +{(\updelta}^{11}{{\text{B}}}_{\text{sw}}-{1000(\upalpha}_{\text{B}}-{1)}{)}{1}{{0}}^{{{\text{p}}{\text{K}}}_{\text{B}}^{*}-{\text{pH}}}}{{1}+{\upalpha}_{\text{B}}{1}{{0}}^{{{\text{p}}{\text{K}}}_{\text{B}}^{*}-{\text{pH}}}}$$

where δ^11^B_sw_ (39.61‰) is the δ^11^B of seawater^68^, αB (1.027) is the fractionation factor between boric acid and the borate ion^69^, and p*K*_B_^*^ is the dissociation constant of the two boron species calculated using site specific temperature, salinity, and pressure estimates (8.83 at 3 °C, 35 psu, and 700 m respectively, typical of sites in this study). Factoring for variation in temperature and salinity on p*K*_B_^*^, estimates of seawater pH uncertainty outlined above correspond to uncertainties on δ^11^B_borate_ that are typically better than ± 0.15‰ (2 SD; maximum ± 0.57‰).

### Estimation of internal Ω_Aragonite_

Inorganic aragonite precipitation experiments show that B/Ca ratios are most strongly correlated with either (i) [B]/[DIC] or (ii) [B(OH)_4_^−^]/[CO_3_^2–^] of the CF^49^. Previous biocalcification models of scleractinian corals have therefore used the theoretical relationship between coral B/Ca and [CO_3_^2–^] of the CF to obtain a second carbonate system parameter using Eqs. (5) and Eq. (6)^37,38,49^:

5 $${[}{\mathrm{CO}}_{3}^{2-}{]}_{\mathrm{CF}}={D}_{\mathrm{B}/\mathrm{Ca }}\frac{{[}{\mathrm{B}(\mathrm{OH})}_{4}^{-}{]}_{\mathrm{CF}}}{{\mathrm{B}/\mathrm{Ca}}_{\mathrm{Aragonite}}}$$

6 $${[}{\mathrm{B}(\mathrm{OH})}_{4}^{-}{]}_{\mathrm{CF}}=\frac{{\mathrm{B}}_{\mathrm{T}}}{{1+[{\mathrm{H}}^{+}{]}_{\mathrm{CF}}{K}_{B}^{*}}}$$

where *D*_B/Ca_ is the partition coefficient of boron into aragonite from seawater, B_T_ is the total boron concentration in seawater (432.5 µmol/kg at salinity 35 psu^68^) and [H^+^]_CF_ is calculated using coral δ^11^B derived pH estimates of the CF. We apply this model to aragonitic stylasterid δ^11^B and B/Ca data and convert [CO_3_^2−^]_CF_ to Ω_Aragonite_ assuming the [Ca^2+^]_CF_ is equal to that of ambient seawater (10.3 mmol/kg). Uncertainty on [CO_3_^2−^]_CF_ is calculated by 1000 Monte Carlo iterations that incorporate analytical uncertainty on δ^11^B and B/Ca measurements and seawater temperature uncertainty based on proximal hydrographic data.

### Coral sample preparation

Coral samples for this study were air dried following collection. The majority of organic matter was removed through both physical scraping and treatment in dilute NaClO for 12 h. Cross sectional discs (~ 2 mm thickness) were cut using a rotary cutting tool from the central trunk (or widest branch) of stylasterid samples. Microstructures within deep-sea corals are known to exhibit contrasting boron concentration^47^ and isotopic composition^43^. Similarly, the δ^18^O composition of growing tips of stylasterid branches has been shown to be further from seawater equilibrium than bulk samples^28^, hence apical tips were avoided and large samples were cut (~ 50 mg; i.e. × 10 mass required for analysis) to diminish the influence of a single microstructural component. To confirm this, corals were also sampled and analysed in duplicate to characterise variability between solid pieces cut from the same coral.

Coral fragments were finely crushed using a pestle and mortar before 5 to 10 mg of the homogenous powder was taken. Residual organic matter was removed using warm 1% H_2_O_2_ (80 °C; buffered in NH_4_OH) and a weak acid leach (0.0005 M HNO_3_) before powders were dissolved in distilled 0.5 M HNO_3_.

### Analytical techniques

All analyses were performed at the University of Bristol. An aliquot of the dissolved sample was analysed by ICP-MS using well-characterised, matrix-matched, synthetic standard solutions to give B/Ca and U/Ca ratios. Samples and standards were introduced in 0.5 M HNO_3_ and a 0.5 M HNO_3_ and 0.3 M HF acid wash solution was utilised between samples/standards to aid B wash out^31^. Repeat analysis of NIST RM 8301 (Coral) yielded mean B/Ca and U/Ca ratios of 527 ± 2% and 822 ± 1% µmol/mol (RSD; n = 35) which are within analytical uncertainty of the interlaboratory consensus value for this reference material^70^.

An aliquot of the dissolved sample containing ~ 25 ng B was separated from the carbonate matrix using 20 μl micro-columns containing Amberlite IRA 743 boron-specific anionic exchange resin^71^. The δ^11^B of purified boron samples were measured by Multi-Collector ICP-MS against NIST SRM 951^35,71^. In this case, all samples, blanks, and standard solutions were introduced to the instrument in a 0.5 M HNO_3_ and 0.3 M HF acid matrix again to ensure optimal B wash out^31^. Full procedural uncertainty was assessed using repeat measurement of NIST RM 8301 (Coral) yielding an average δ^11^B value of 24.27 ± 0.16‰ (n = 25; 2* s*) which again was within uncertainty of interlaboratory consensus values^70^. Total procedural blanks (n = 6) were small (less than 39 ng of boron) and were thus < 0.2% of the boron loaded onto columns for samples.

## Supplementary Information

Supplementary Information 1.

Supplementary Information 2.

## Data Availability

All new boron isotope (δ^11^B), B/Ca, and U/Ca data from stylasterid corals in this study are included in Supplementary Information. These tables also include coral species and location information and matched proximal hydrographic data.
