# Supplementary material for: Stylasterid corals build aragonite skeletons in undersaturated water despite low pH at the site of calcification
Source: Sci Rep. 2022 Jul 30;12:13105. doi: 10.1038/s41598-022-16787-y (PMC9339005; doi:10.1038/s41598-022-16787-y)
Supplement: Supplementary file 2 — Supplementary Information 2. [file 41598_2022_16787_MOESM2_ESM.docx]

**Stylasterid corals build aragonite skeletons in undersaturated water despite low pH at the site of calcification**

**Joseph A. Stewart** ^a*^**, Ivo Strawson** ^a,b^**, James Kershaw** ^a^**, Laura F. Robinson** ^a^

^a^ School of Earth Sci. Univ. of Bristol, Queens Road, Bristol, BS8 1RJ, UK

^b^ Department of Earth Sciences, University of Cambridge, Downing Street, Cambridge CB2 3EQ, UK

*correspondence [joseph.stewart@bristol.ac.uk](mailto:joseph.stewart@bristol.ac.uk)

### Supplementary information

### *Stylaster ibericus*

Compared to other pure aragonitic stylasterids in this study the single *Stylaster ibericus* specimen that lived under the highest pH conditions (8.04; located in the Northeast Atlantic) gave anomalous results. These included low average B/Ca ratios (477 µmol/mol), highly variable δ^11^B between replicate sections of the coral (range > 2 ‰), and high overall mean skeletal δ^11^B (18.8 ‰) that was + 2.3 ‰ higher than our estimates of δ^11^B_borate_ for that site. In linear regression analysis of skeletal δ^11^B against δ^11^B_borate_ this sample yielded a high Cook’s distance (> 4/*n*, where *n* is the number of observations) further identifying it as a potential outlier.

After rigorous oxidative cleaning had been applied to samples (both air-drying and bleaching of whole specimens and peroxide cleaning of powders; see methods), we consider it unlikely poor reproducibility in the *Stylaster ibericus* sample stems from varying amounts of residual organic matter. Uncertainty in hydrographic conditions at this site (±0.08 pH units; 2 SD) also cannot sufficiently explain such high overall δ^11^B and B/Ca values approximately 50% lower than other aragonitic stylasterid genera. The low B content of this specimen is more similar to high-Mg calcitic stylasterids, yet there are many reasons why we rule out incorporation of a significant high-Mg calcite phase. X-ray diffraction analysis ^1^ and generally low Mg content (3.1 µmol/mol; ^2^) of this specimen both suggest a 100% aragonitic mineralogy of this *Stylaster ibericus* specimen, confirming that this was not a mis-identified mixed mineralogy specimen (e.g. *E. gracilis*). All corals in this study were either collected alive or were visibly well-preserved, however we cannot fully discount the influence of an unseen contaminant carbonate phase (e.g. burrowing organisms or epiphytes) or early diagenesis on this *Stylaster ibericus* sample. Despite this clearly anomalous behaviour, in the absence of other individuals of this taxon, it is impossible to assess whether these observations are unique to this specimen or all *Stylaster ibericus*. Thus, while we find no reason to exclude this specimen inclusion of this sample has little bearing on the general patterns of skeletal chemistry and findings described here.

### Potential utility of stylasterids as a paleo-pH biosensor

The boron isotope proxy can give both insight into biocalcification mechanisms and offer records of past seawater pH from fossil coral individuals ^3,4^ provided the δ^11^B sensitivity to ambient seawater pH in the organism has been suitably calibrated across a range of modern environmental conditions ^5^.

Solid replicate cross-sectional discs cut from the same coral using the protocol outlined here yield trace element and isotopic results that replicate well, suggests that stylasterid corals lack the strong microstructural heterogeneity of some scleractinia (e.g. δ^11^B differences of up to 8 ‰ between centres of calcification and fibrous aragonite in *Lophelia pertusa*; ^6^). Good reproducibility of stylasterid measurements potentially bodes well for their use in paleoceanographic proxy reconstructions (e.g. ^1,2^) and suggests that labour-intensive microsampling is likely unnecessary for this taxon (*c.f.* scleractinian corals; ^6,7^).

Linear regression of stylasterid δ^11^B measurements against δ^11^B_borate_ are discussed in the main text and yield following relationships for all stylasterid δ^11^B measurements (regardless of mineralogy; Equation 1), aragonitic stylasterids only (Equation 2), and weighted regression of aragonitic stylasterids (Equation 3), against δ^11^B_borate_ (SE):

δ^11^B_Stylasterids All_ = 1.28 (0.33) × δ^11^B_borate_ – 4.10 (4.96) [R^2^ = 0.43]

Equation 1

δ^11^B_Stylasterid Arag._ = 1.57 (0.33) × δ^11^B_borate_ – 8.66 (5.05) [R^2^ = 0.62]

Equation 2

δ^11^B_Stylasterid Arag. WT_ = 1.04 (0.29) × δ^11^B_borate_ – 0.72 (4.34) [R^2^ = 0.49]

Equation 3

The regression in Equation 3 weights observations according to the inverse of the uncertainty on their mean skeletal δ^11^B measurements.

While the two high-Mg calcitic stylasterid corals in this study exhibit δ^11^B values only slightly higher (+1.5 ‰) than values predicted for δ^11^B_borate_ in seawater, without further individuals from a wide range of pH conditions the sensitivity of high-Mg calcitic specimens to seawater pH and significance of any offset from δ^11^B_borate_ remains unclear. We do however have sufficient aragonitic specimens to demonstrate that stylasterid skeletal δ^11^B varies as a function of the δ^11^B_borate_ of ambient seawater. The linear regression using all stylasterids, regardless of species or mineralogy, yields an R^2^ value of 0.43 (Equation 1). The variance on this calibration is even lower if solely aragonitic stylasterids are selected and the regression is weighted to account for uncertainty in the *y*-axis (R^2^ = 0.49), thus diminishing the influence of the *Stylaster ibericus* specimen that did not reproduce well compared to other specimens. The variance on these aragonitic stylasterid regression lines is similar to that of the well-characterised cold-water scleractinian *D. dianthus* that has been used to characterise past seawater pH and carbon cycling during the last deglaciation ^3,4^. Unlike the monospecific *D. dianthus* regression however, our calibration includes a mix of aragonitic stylasterid species making this result all the more encouraging. This raises the strong possibility for the use of stylasterid coral δ^11^B as a monitor of paleo-pH of seawater particularly if calibrations can be refined to a single species.

**References**

1 Samperiz, A. *et al.* Stylasterid corals: A new paleotemperature archive. *Earth and Planetary Science Letters* **545**, 116407, (2020).

2 Stewart, J. A. *et al.* Refining trace metal temperature proxies in cold-water scleractinian and stylasterid corals. *Earth and Planetary Science Letters* **545**, 116412, (2020).

3 Stewart, J. A. *et al.* Productivity and Dissolved Oxygen Controls on the Southern Ocean Deep-Sea Benthos During the Antarctic Cold Reversal. *Paleoceanography and Paleoclimatology* **36**, e2021PA004288, (2021).

4 Rae, J. W. B. *et al.* CO_2_ storage and release in the deep Southern Ocean on millennial to centennial timescales. *Nature* **562**, 569-573, (2018).

5 McCulloch, M. *et al.* Resilience of cold-water scleractinian corals to ocean acidification: Boron isotopic systematics of pH and saturation state up-regulation. *Geochimica et Cosmochimica Acta* **87**, 21-34, (2012).

6 Blamart, D. *et al.* Correlation of boron isotopic composition with ultrastructure in the deep-sea coral *Lophelia pertusa*: Implications for biomineralization and paleo-pH. *Geochemistry, Geophysics, Geosystems* **8**, Q12001, (2007).

7 Stewart, J. A., Anagnostou, E. & Foster, G. L. An improved boron isotope pH proxy calibration for the deep-sea coral *Desmophyllum dianthus* through sub-sampling of fibrous aragonite. *Chemical Geology* **447**, 148-160, (2016).
